# Effect of devulcanized reclaimed rubber content on structure, mechanical properties, and thermo-oxidative aging behavior of ethylene-propylene-dien-monomer (EPDM) rubber

**DOI:** 10.1038/s41598-026-36961-w

**Published:** 2026-02-11

**Authors:** Yuchen Leng, Vanessa Spanheimer, Danka Katrakova-Krüger, Ulrich Giese

**Affiliations:** 1 Materials Laboratory , TH Köln - University of Applied Sciences , Steinmüllerallee 1, 51643 Gummersbach, Germany; 2 German Institute for rubber technology, Eupener Straße 33, 30519 Hannover, Germany

**Keywords:** Devulcanization, Crosslinking, Rubber recycling, Recipe adjustment, Thermo-oxidative aging, Chemistry, Engineering, Materials science

## Abstract

Rubber recycling refers to the circular economy process of processing waste rubber products into reclaim or rubber crumb/granulates through methods such as chemical treatment (e.g., devulcanization) and grinding. Especially the devulcanization has the goal of restoring its plasticity for reuse and revulcanization. However, the recycling process degrades rubber quality and incurs high energy consumption and costs, limiting its application. This study investigates the effects of varying concentrations of devulcanized EPDM on the rheology, mechanical properties, and thermo-oxidative aging behavior of EPDM compounds. Devulcanized rubber obtained through selective cracking of sulfur crosslinks was incorporated into an EPDM compound at different ratios. The addition of devulcanized EPDM accelerates the curing, but also reduces the crosslink density and the network uniformity. Mechanical testing indicates that higher devulcanized EPDM content increases shore A hardness but decreases tensile strength and elongation, attributed to insufficient interaction to the new rubber matrix, residual crosslinks and filler content in the recycled rubber. Thermo-oxidative aging evaluations indicate that devulcanized EPDM does not accelerate degradation or embrittlement, generally matching or even lagging behind the reference material. Furthermore, recipe adjustment can moderately improve properties of devulcanized EPDM, such as crosslink density and flexibility. These findings provide valuable insights for optimizing recycled rubber formulations, balancing sustainability with performance requirements.

## Introduction

The widespread application of rubber materials hinges on their intriguing thermomechanical properties, particularly their high elasticity and damping characteristics. Elastomers undergo a high-temperature vulcanization process to develop these properties. During this process, polymer chains are chemically bonded to form primary crosslinks, creating a three-dimensional molecular network^1^. Consequently, rubber recycling poses significant challenges, and devulcanization stands as a critical process in reclaiming rubber materials. Devulcanization is defined as “the complete or partial breaking of polysulfidic (-Sx-), disulfidic (S-S), and monosulfidic (C-S) cross-link bonds formed during the initial vulcanization process in vulcanized rubber”^2^ without damaging the backbone network or degrading the material properties. Devulcanized rubber can be blended with virgin rubber or other types of matrices to form new compounds without significantly compromising mechanical and physical characteristics^3^.

Typical devulcanization processes (during which the rubber network structure is destroyed) can be categorized into five types: chemical processes, mechanical processes, thermochemical processes, radiation processes, and biological processes. The chemical process involves combining rubber waste powder with chemical agents such as dialkyl-or diarylsulfides, thiophenols and its zinc salts, and mercaptan compounds to disrupt the rubber crosslinking network. Most chemical devulcanization methods are batch-based, with the drawback of involving toxic substances^2^. Mechanical processes achieve rubber network rupture solely through shear forces and can be implemented using twin-roll mills, batch mixers, or single/twin-screw extruders^4^. Thermochemical processes combine heat with reactivators to break crosslinking points, typically conducted in high-pressure internal batch mixers or extruders^5^. Radiation processes, including microwave^6^ and ultrasonic^7^ devulcanization, can disrupt the three-dimensional network structure of rubber. From an environmental perspective, biological processes (microbial metabolism) represent an effective means of devulcanization, selectively breaking the sulfur bonds in vulcanized rubber through microbial bio-attack. However, devulcanization occurs only at the rubber surface, proceeding slowly, time-consuming, and with low conversion efficiency^8^. Extrusion-based thermomechanical devulcanization appears more suitable for industrial-scale application^3^.

Devulcanization process facilitates the recovery of rubber polymers, enabling their reuse in various applications, reducing waste, and promoting sustainable development. However, the incorporation of devulcanized rubber affects the mechanical, thermal and dynamic properties of the final material, though this is also dependent on the devulcanization process and parameters^9^. Research indicates that devulcanized rubber can partially restore elasticity and processability, but mechanical strength often depends on devulcanization efficiency and compatibility with the base polymer. For instance, studies suggest that increasing devulcanized rubber content reduces the vulcanization characteristics of the mixture, leading to decreases in tensile strength, elongation at break, tear strength, and rebound elasticity, while increasing Shore A hardness, tensile modulus, and stiffness^10^. This mechanical degradation correlates with failure points identified via surface fracture imaging. Incomplete devulcanization and residual crosslink density restrict chain mobility, resulting in increased material hardness^11^. Conversely, optimizing devulcanization processes and incorporating compatibilizers may enhance interfacial adhesion and mechanical properties in vulcanized compounds. Thermally, devulcanized rubber exhibits good stability and is suitable for reuse. However, changes in the rubber network structure may cause recycled compounds to display altered dynamical properties and even strain-induced crystallization behavior^12^. Higher proportions of devulcanized rubber can increase the glass transition temperature (Tg) and storage modulus (E’), while simultaneously reducing the loss factor (tan δ)^13^. Under optimized process parameters and reduced expectations on physical properties, recycled rubber can replace up to approximately 65 wt% of virgin natural rubber^14^. Incorporating 40 wt% of virgin rubber with devulcanized rubber into the compound does not adversely affect scorch or optimal vulcanization time, nor does it significantly degrade the mechanical properties of the virgin rubber^15^. Recent studies indicate that batch devulcanization at recycled rubber contents as high as 50 wt% results in only a slight decline in product quality, maintaining tensile strength while exhibiting a minor decrease in modulus^16^.

Beyond the aforementioned properties, material durability is particularly crucial in industrial applications. However, devulcanized rubber undergoes aging during storage and subsequent use, significantly impairing its performance. Depending on the severity of exposure, both physical and chemical aging may occur simultaneously. The former causes mechanical degradation, including loss of elasticity, swelling, and internal stress relaxation, while the latter leads to irreversible chemical changes at the polymer chains and in the polymer network^17^. Among various aging processes, thermal oxidation stands as one of the most prevalent phenomena^18^. It refers to the degradation process occurring in rubber materials when exposed to prolonged high temperatures and oxygen environments, subsequently altering the material’s physical and mechanical properties. This degradation is induced by changes in the chemical structure of the rubber material, involving two primary competing mechanisms: crosslinking and chain scission^19^. Aging alters crosslink density and network structure, leading to further re-crosslinking or additional crosslink formation. This changes the rubber’s molecular structure, increasing crosslink density and material hardness. Conversely, polymer chains may also undergo breakage and degradation, reducing molecular weight and mechanical integrity^20^. Chain scission in rubber vulcanizates during oxidation occurs as a side reaction, maintaining a nearly constant ratio relative to the main reaction, crosslinking, thereby elevating overall crosslink density^21^. However, some studies suggest crosslinking occurs throughout the aging process, while chain scission occurs later and competes with crosslinking. Crosslink density increases at a nearly constant rate, slowing in the later stages, and is closely correlated with hardness and stiffness^22^. During aging, the amount of water adsorbed by the polymer increases. This phenomenon also stems from the formation of hydrophilic functional groups, which enhance the polymer’s affinity for water^23^.

To further enhance the application benefits of devulcanized in rubber recycling, this study investigated the properties of compounds containing devulcanized ethylene propylene diene (EPDM) rubber at varying concentrations. EPDM is a terpolymer formed by the copolymerization of ethylene, propylene, and diene monomers, with the diene monomers introducing unsaturated bonds or double bonds into the polymer chain for sulfur vulcanization. Currently, EPDM is the fastest-growing general-purpose rubber material, owing to its outstanding properties—particularly its exceptional ozone resistance and ability to withstand high solid filler loading^24^. Concurrently, recipe adjustments were made to compensate for further ingredients in the devulcanized material by reducing carbon black and softener content. Although EPDM is rather aging resistant the use of a secondary raw material like the devulcanized rubber may lead to unknown and unforeseen reactions over time due to therein contained (varying) impurities. Therefore, additionally, the thermo-oxidative aging behavior of the compounds with devulcanized EPDM rubber were examined in comparison to the reference compound through a 6-week aging process conducted in air at three distinct temperatures. The influence of the recycled rubber content and recipe adjustment on the compound properties and oxidative behavior is critical for subsequent industrial applications.

## Materials and methods

### Compounding

Devulcanized EPDM rubber ReMould EPDM from J. Allcock and Sons was used in this study. Its composition, is given as in Table 1. According to the manufacturer, it is produced by selectively breaking sulfur-sulfur bonds using chemicals and mechanical force. They also declare that during this process, the polymer chains are not damaged and the crosslink density is reduced by around 90%.

**Table 1:** Manufacturer specification: composition of remould EPDM.

Substance	wt%
Polymer component (EPDM)	~ 30
Carbon black	~ 30
Rubber process oils	~ 10
Fillers	~ 30

The model recipe for a sealing compound based on ethylene propylene diene rubber (EPDM) is shown in Table 2. All measurements were done on the vulcanizates of the compounds without (as reference) and with the respective amount of devulcanized rubber (20%, 40%, and 60% Devulcanized EPDM). The used EPDM was Keltan^®^ 8550 ECO from ARLANXEO with 70% bio-based content. It is amorphous with a medium ethylidene norbornene (ENB) content of around 5%. The polymer was partially substituted with the devulcanized EPDM in 20 phr (parts per hundred rubber) steps, regarding the polymer content of the devulcanized material measured by thermogravimetric analysis (TGA). The phr amount of the polymer and the polymer content of the devulcanized EPDM adds up to 100 phr. For the 60% substitution, adjustment was made to the recipe regarding not only the polymer content, but also the softener and carbon black content to reduce them as well.

**Table 2:** Model compound recipe (unit: phr).

	Reference	20% Devulcanized EPDM	40% Devulcanized EPDM	60% Devulcanized EPDM	60% Devulcanized EPDM with recipe adjustments
EPDM (Keltan 8550 ECO)	100	80	60	40	40
Devulcanized EPDM	0	55	110	165	165
Carbon black N550	110	110	110	110	61
Calcium Carbonate (CaCO_3_)	80	80	80	80	80
Zinc oxide (ZnO)	3	3	3	3	3
Polyethelene glycol (PEG 4000)	2	2	2	2	2
Stearic acid	0.5	0.5	0.5	0.5	0.5
Softener	85	85	85	85	77
Calcium oxide (CaO)	8	8	8	8	8
Sulfur (S)	0.8	0.8	0.8	0.8	0.8
Accelerators	4.2	4.2	4.2	4.2	4.2
Retarder	0.1	0.1	0.1	0.1	0.1

The compounds were processed in two stages using a Werner and Pfleiderer GK 1.5 E internal mixer. Both stages were conducted at an initial temperature of 60 °C and a mixing speed of 50 rpm (revolutions per minute). During the first stage, all components except sulfur, accelerators, and the retarder were added to the mixer. The mixing process comprised several steps: an initial 6-minute mixing period followed by ventilation, then an additional mixing period to reach a total of 8 min, another ventilation step, and finally, a third mixing period bringing the total time to 10 min. The second stage took place on the following day. Initially, the masterbatch was mixed for 1 min. Subsequently, the crosslinking system was added and the mixture was mixed for additional 3 min. After mixing, 2-mm-thick test slabs were prepared and vulcanized. This process was carried out at 180 °C for 8 min under a pressure of 150 bar, using a laboratory press (Gibitre Instruments S.r.l.).

### Raw material characterization

The quantitative determination of free sulfur content in rubber samples is achieved by measuring sulfur content before and after Soxhlet extraction. Dichloromethane is used as the extraction solvent, effectively removing soluble sulfur compounds and other extractable components from the sample. Following extraction, the residue is incinerated at 1350 °C in an oxygen-enriched environment. High-temperature oxidation converts all sulfur in the sample into sulfur dioxide (SO₂) gas. The generated SO₂ is captured using a dilute sulfuric acid solution containing hydrogen peroxide to ensure complete oxidation and stabilization of sulfur compounds in the liquid phase. This reaction induces a measurable change in the conductivity of the acid solution, providing a sensitive and relative conductometric method for sulfur content determination. According to^24^, the magnitude of the conductivity change is proportional to the sulfur content in the original sample, serving as a reliable surrogate indicator for sulfur content. The measured sulfur content represents the total sulfur content, encompassing not only sulfur from fracture crosslinking agents but also the total sulfur content contributed by fillers, oils, and any potential accelerator residues. Beyond sulfur analysis, the overall material composition of the samples was evaluated via thermogravimetric analysis (TGA). TGA measurements were conducted in two distinct steps, each employing specific temperature and atmosphere conditions (detailed in Table 3). This two-step approach distinguishes thermal degradation stages corresponding to different components within the sample—such as polymers, fillers, and additives—thereby providing a comprehensive compositional profile of the material.

**Table 3:** TGA measurement parameters using analyzer STA 409PC/PG (Netzsch, Germany).

	First step	Second step
Temperature program	Heating from 30 to 600 °C	Heating from 600 to 900 °C
Heating rate	10 K/min	10 K/min
Gas flow	30 ml/min N_2_	20 ml/min N_2_ + 10 ml/min O_2_

### Compound characterization

Using the Rubber Process Analyzer (RPA Flex, TA Instruments), curing tests were conducted to measure the viscoelastic properties of a rubber compound during vulcanization (curing) at a controlled temperature of 180 °C for 10 min. The Payne effect was also measured at 100 °C to understand the behavior of the filler network, as well as the viscosity and flow. TSSR is an experimental technique that characterizes the viscoelastic behavior, crosslink density, and aging-related changes of polymeric materials, including elastomers, by measuring their stress relaxation response under controlled temperature gradients. The sample, approximately 2.2 millimeters thick, was heated from room temperature (23 °C) to 300 °C at a heating rate of 0.033 K/min, with an isothermal relaxation time of two hours. Crosslink density was assessed via equilibrium swelling in cyclohexane (Carl Roth), and anisothermal relaxation behavior was analyzed using a Temperature-Scanning-Stress-Relaxation Meter (TSSR, Brabender) from 23 to 300 °C according to ASTM D8363-20.

Mechanical properties were evaluated using standardized specimens, including the tensile strength of S2 dumbbells and the tear resistance of Graves angle test pieces. These evaluations were performed in accordance with DIN 53,504 and DIN ISO 34 − 1, respectively, using a 10 kN allround tabletop universal testing machine (Zwick Roell). The compression set was determined following DIN ISO 815 after aging for 22 h at 100 °C in a Heraeus T6060 oven. Shore A hardness measurements were performed on stacked specimens with a total thickness of 6 mm according to DIN ISO 48 − 4 using a hardness tester from Karl Frank GmbH. Experiments were repeated on 5 samples.

All compounds underwent accelerated aging at three different temperatures and durations in the Heraeus oven, as detailed in Table 4. The aging times at 125 °C were estimated based on the principle that a 10 K increase in temperature halves the required reaction time. This correlation was used to equate the aging times at 125 °C to equivalent times at 100 °C. Subsequently, mechanical tests were performed on all aged materials to evaluate changes in performance.

**Table 4:** Applied aging states.

Temperature	Duration		
70 °C	1 week	3 weeks	6 weeks
100 °C	1 week	3 weeks	6 weeks
125 °C	29.7 h	89.1 h	178.2 h

Two additional methods were employed to evaluate crosslinking changes during aging. First, the freezing point depression of cyclohexane was measured in the reference compound and in compounds containing 60% devulcanized EPDM, with and without recipe adjustments. The freezing point depression, which is the difference between the freezing point of the pure solvent and the trapped solvent, can be measured to determine the crosslink distance. Crosslinking distance is inversely proportional to crosslinking density. Differences in crosslinking quantity affect the size of the solvent’s mobile region, thereby influencing the freezing temperature^25^. Measurements were performed on unaged samples and on samples aged for the longest duration across all tested temperatures using a Netzsch DSC 200 PC (see Table 5 for measurement parameters).

**Table 5:** DSC measurement parameters for freezing point depression.

Heating/cooling rate	5 K/min
Gas flow	70 ml/min N_2_
Temperature program	1. Cooling from 25 to − 50 °C 2. Heating from − 50 to 25 °C

Second, a low-field proton nuclear magnetic resonance (¹H NMR) analysis was conducted on the same set of samples: the reference sample and the 60% devulcanized EPDM compounds with and without recipe adjustments, in both the unaged state and after the maximum aging period (178.2 h at 125 °C). The binding force of H proton in each chain is decreased in turn and the T2 relaxation time reflects the degree of freedom of each chain and the crosslink density as well^26^. The NMR measurements were carried out using a Bruker minispec mq20 spectrometer operating at a resonance frequency of 20 MHz. All analyses were conducted at a controlled temperature of 90 °C to ensure consistency.

## Results and discussion

### Raw material characterization

The devulcanized EPDM contains 1.42% free sulfur, which can influence the crosslinking behavior and the resulting network structure of newly formulated materials.

**Fig. 1:**
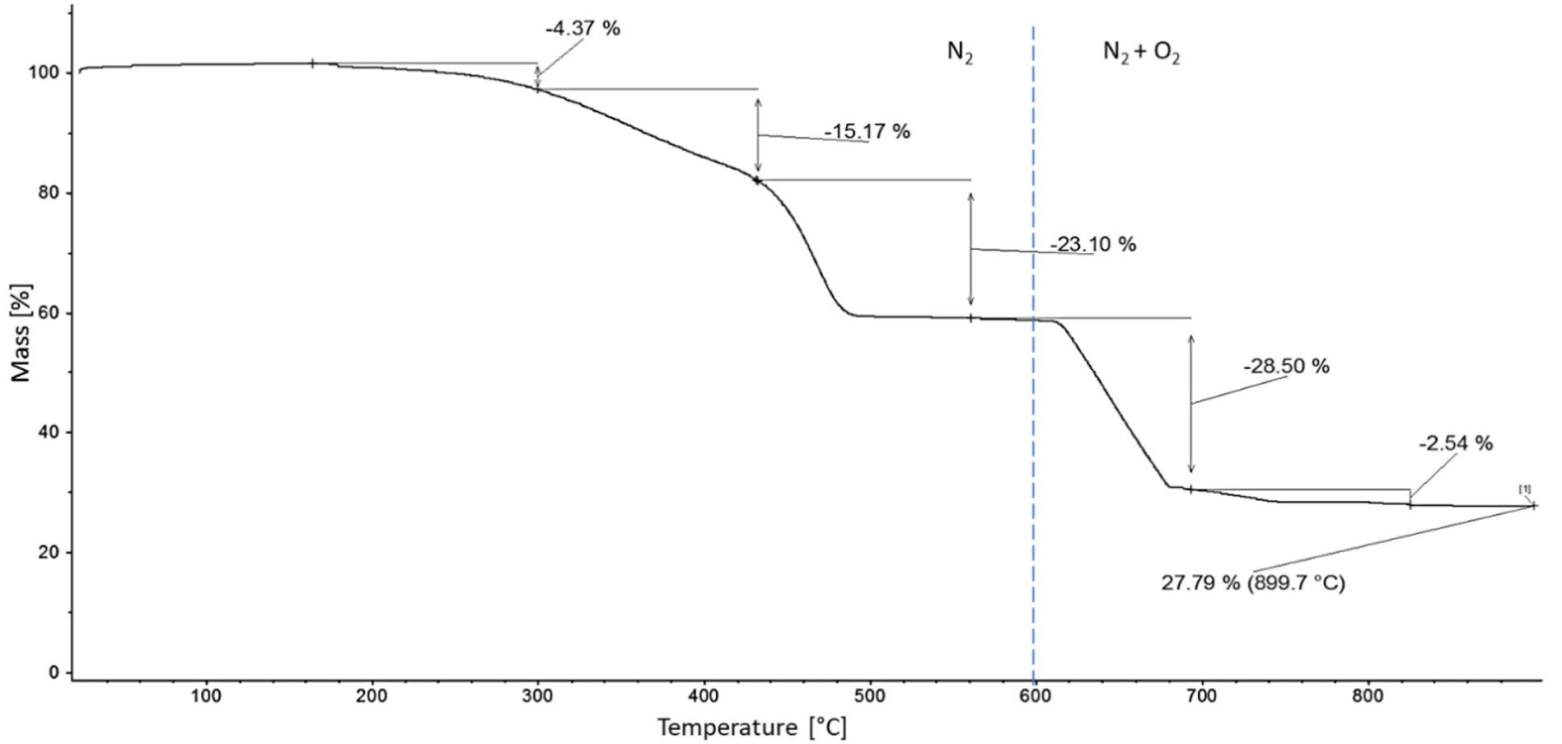
TGA curve of the devulcanized EPDM.

TGA results of the devulcanized EPDM showed mass loss and residual mass values (see Fig. 1; Table 6) consistent with the data reported in the safety data sheet by J-Allcock & Sons Ltd. The significant presence of carbon black and other mineral fillers contributes to increased stiffness in the resulting compounds. To maintain comparable mechanical properties, the formulation with the highest devulcanized EPDM content was adjusted to preserve the original filler and oil ratios of the recipe.

**Table 6:** TGA mass losses and residual mass.

Temperature range	Mass loss [%]	
25–300 °C	4.37	Volatile compounds, softener
300–430 °C	15.17	Softener, shortened polymer chains due to chain scission
430–560 °C	23.10	Polymer
560–690 °C	28.50	Carbon black
690–820 °C	2.54	CO_2_ from calcium carbonate (CaCO_3_) scission CaCO_3_ content can be calculated by stoechiometric factor appr. 2.5
Residual mass at 900 °C	27.79	Mineral content, contains calcium oxide (CaO) from CaCO_3_ degradation

### Compound characterization

#### Curing

**Fig. 2:**
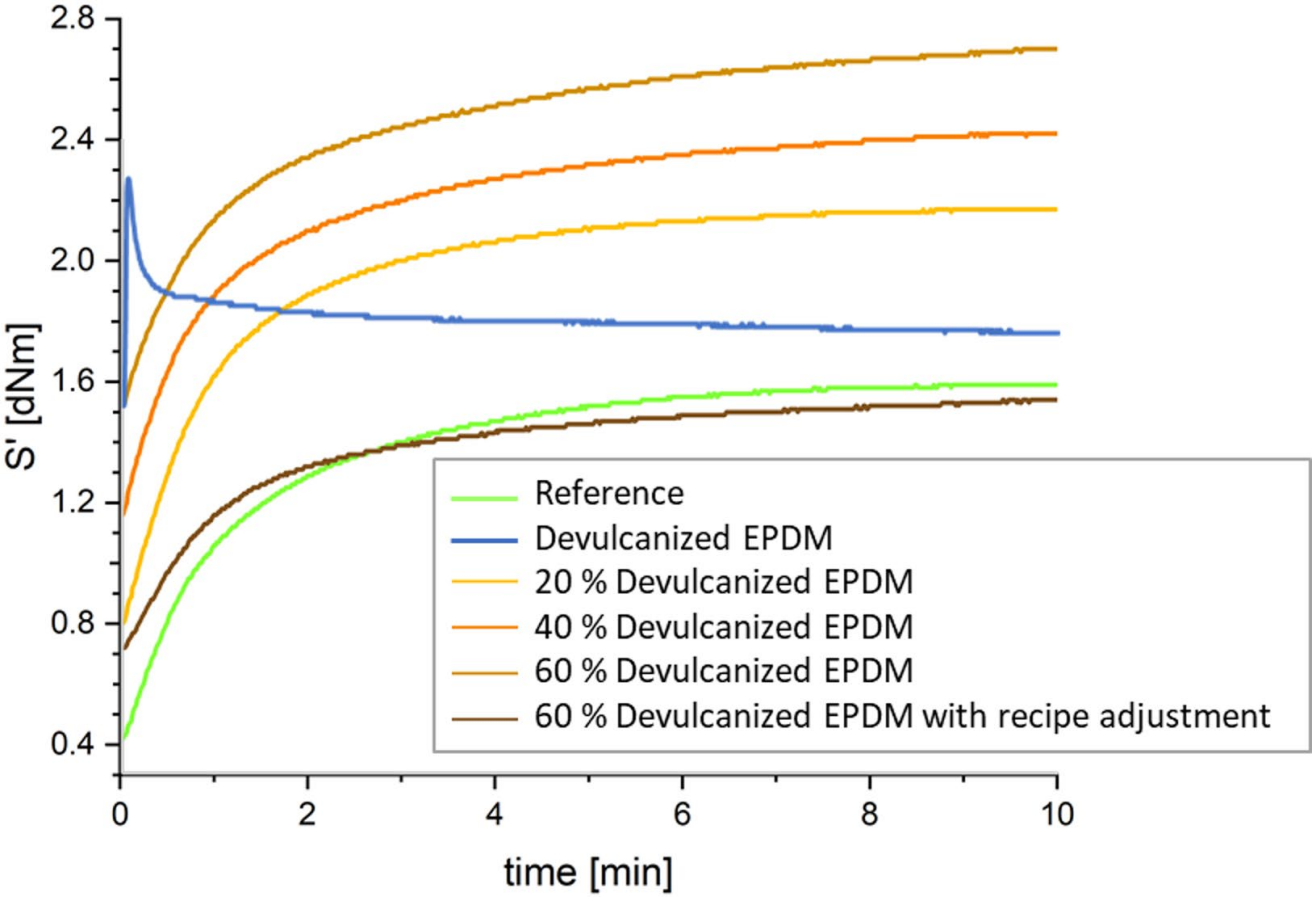
Curing curves of all rubber compounds at 180 °C.

Figure 2 shows the rheometer curves of pure devulcanized EPDM and composites with various concentrations at 180 °C while Table 7 displays their curing characteristics. The blue curve represents devulcanized EPDM, exhibiting a significantly higher maximum torque value after curing compared to the original natural rubber. This indicates that the polymer chains of devulcanized EPDM rubber have not degraded; instead, the crosslinked network has been selectively disrupted and can be reconstituted. Torque tends to decrease slowly at the end of the analysis, a phenomenon likely resulting from the gradual degradation of the rubber backbone following prolonged microwave irradiation. As the content of devulcanized EPDM increases, the initial torque rises gradually, as does the maximum torque (S’_max_), indicating an enhanced overall crosslink density of the rubber network. The increments (ΔS’) are all greater than the reference values, though they slow down as the devulcanized EPDM content increases. One exception is the compound with 60% devulcanized EPDM with recipe adjustment, for which S’_max_ and ΔS’ are both below the reference values. Additionally, the curing reaction rate decreased with increasing devulcanized EPDM content, regardless of recipe adjustment. The average curing time (t_90_) for compounds containing devulcanized EPDM was 2.7 min, outperforming the reference value of 3.33 min. All compounds exhibited plateau-type curing behavior with exception of the 40 and 60% compounds. The show a slightly marching modulus. No reversion is observed for all the others prior to the end of the specified curing time.

**Fig. 3:**
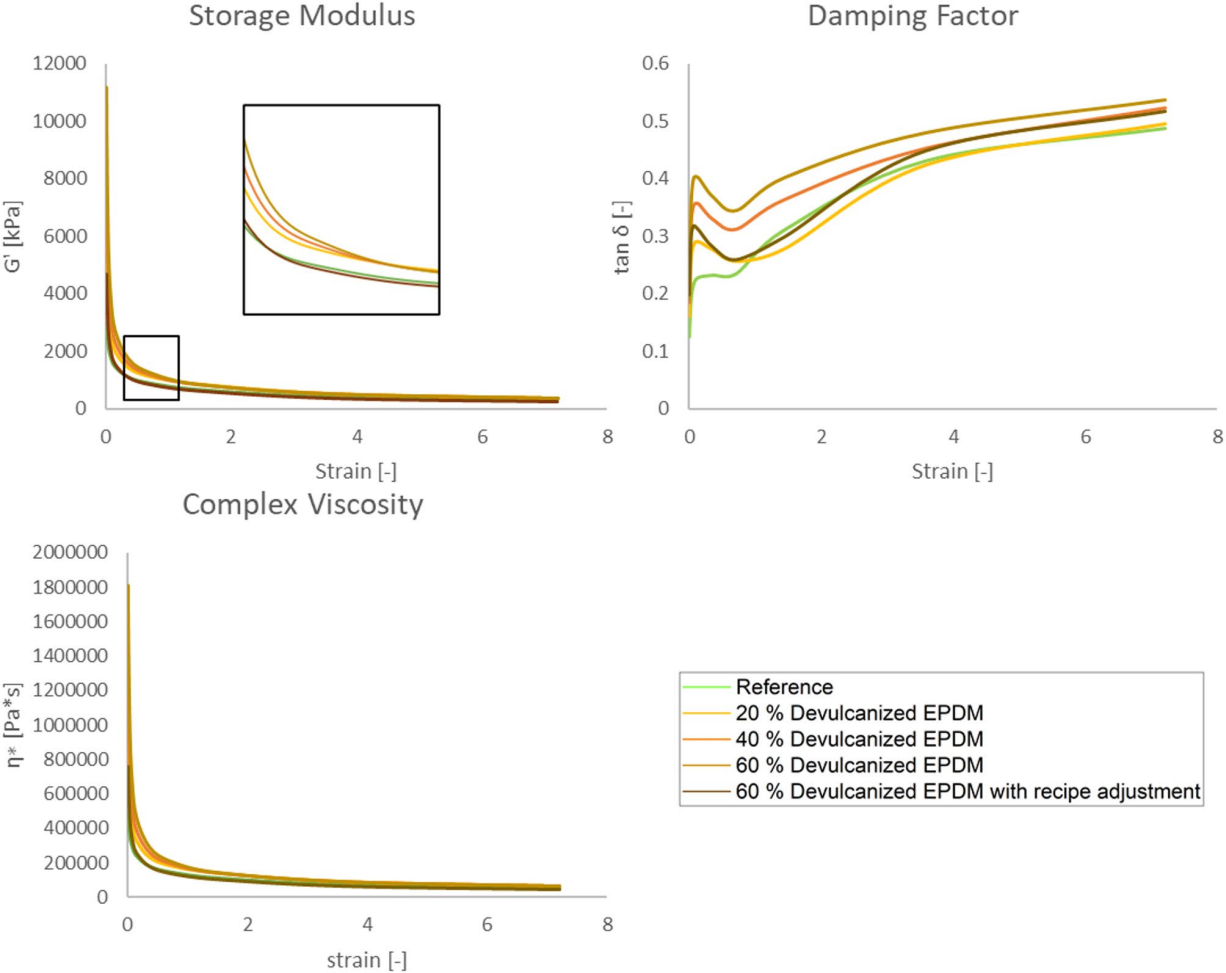
Payne effect: storage modulus (G’), damping factor (tan δ), and complex viscosity (η*) of all rubber compounds at 100 °C.

The decrease in dynamic storage modulus (G’) observed in the Payne effect experiment reflects the disruption and reorganization of the filler-filler network (such as carbon black or other reinforcing particles), as illustrated in Fig. 3. The storage modulus (G’) and complex viscosity (η*) curves of the reference and reformulated compounds are closely aligned, exhibiting a faster decline than other compounds containing devulcanized EPDM. Furthermore, as the proportion of devulcanized EPDM increases, the viscosity behavior of the compounds diverges more significantly from that of the reference material. The loss factor (tan δ) quantifies damping or energy dissipation (the ratio of loss modulus to storage modulus). Within the Payne effect region, the peak tan δ reflects energy dissipation associated with filler interactions and network dynamics—that is, increased material damping due to greater energy consumption from breakdown and friction within the filler network. Higher or broader tan δ peaks typically indicate stronger or more extensive filler network structures.

**Table 7:** Curing properties of all rubber compounds at 180 °C.

Parameter	S’ _max_ [dNm]	∆ S’ [dNm]	t_90_ [min]
Reference	1.59	1.17	3.33
Devulcanized EPDM	2.27	0.75	-
20 % Devulcanized EPDM	2.17	1.37	2.47
40 % Devulcanized EPDM	2.42	1.26	2.68
60 % Devulcanized EPDM	2.7	1.17	2.8
60 % Devulcanized EPDM with recipe adjustment	1.54	0.82	2.87

#### Crosslink density

**Fig. 4:**
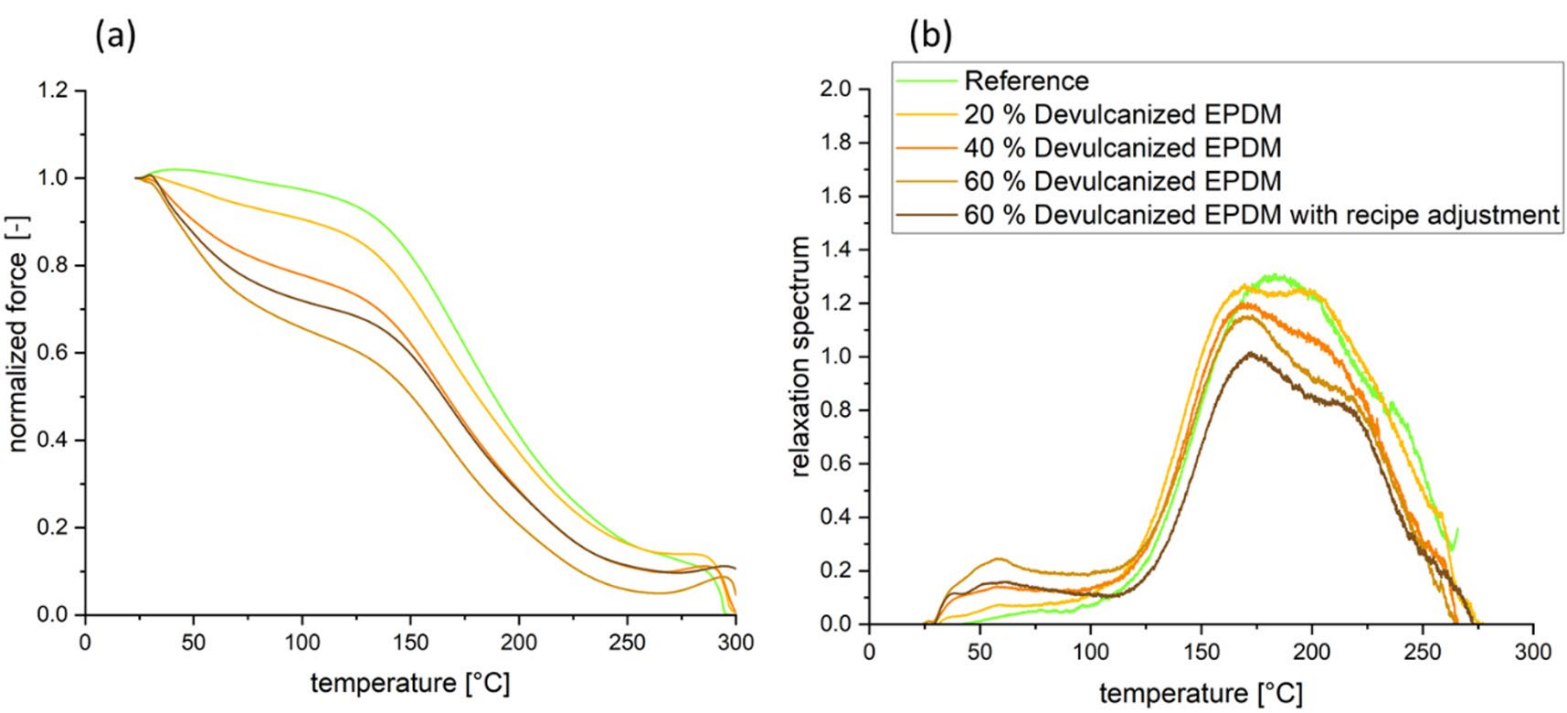
TSSR curves: (**a**) normalized force curves; (**b**) relaxation spectrum curves.

Figure 4 illustrates the effects of devulcanized EPDM content obtained via TSSR on rubber network structure and stress relaxation behavior. Crosslink density, initial stress σ₀, and T_50_ values are listed in Table 8; however, only the crosslink density of the reference material can be determined by the TSSR analyzer routine. The calculation of crosslink density based on TSSR relies on the Gaugh-Joule effect, which describes the shrinkage of rubber when exposed to a heating environment under constant strain^27^. Consequently, this method depends on identifying a distinct plateau modulus at a specific temperature, reflecting the response of the elastic network after the viscoelastic relaxation process has decayed. However, compounds other than the reference fail to yield results because no distinct plateau modulus can be identified in the curve profile (approximately below 100 °C) as shown in Fig. 4b. A possible reason is that samples containing devulcanized EPDM cannot form a homogenious elastomer network, thus preventing the determination of the plateau phase, i.e., the crosslink density.

**Table 8:** TSSR properties of all compounds.

Parameter	Crosslink density [mol/m³]	σ_0_ [MPa]	T_50_ [°C]
Reference	114	0.47	188.6
20% Devulcanized EPDM	n. a.	0.56	180.9
40% Devulcanized EPDM	n. a.	0.55	166.8
60% Devulcanized EPDM	n. a.	0.59	155.6
60% Devulcanized EPDM with recipe adjustment	n. a.	0.48	165.1

Figure 4(a) displays the normalized stress-temperature curves for the samples. Compared to the reference curve, the devulcanized EPDM compound exhibits more pronounced stress relaxation. Between 25 and approximately 40 °C, the stress of the reference curve increases slightly with rising temperature due to the entropy effect. This phenomenon is less pronounced in the other four curves, where yield stress initially compensates for the entropy effect, rendering stress increases imperceptible in this temperature range. Above approximately 130 °C, a sharp stress decay occurs until chemically induced relaxation processes at high temperatures reduce stress to near-zero values. When devulcanized EPDM is present, samples exhibit higher initial stress σ₀. However, the initial stress of the sample containing 60% devulcanized EPDM with recipe adjustments, is comparable to the reference value.

In the relaxation spectrum in Fig. 4(b), the center of the reference peak is located at approximately 185 °C. This peak can be attributed to the breakage of short sulfidic bonds, namely mono- and disulfide crosslinks. Mono- and disulfide crosslinks exhibit higher thermal stability, whereas polysulfide crosslinks undergo chemical degradation at lower temperatures. Compounds containing devulcanized EPDM exhibit a small peak around 60 °C, which indicates the molecular chain relaxation and the physical interaction of non-rubber components. The double peak at 120 °C and within the 210–220 °C range suggests the formation of polysulfide and monosulfide/disulfide crosslink structures, respectively^28^. However, as the content of devulcanized EPDM increases, the double-peak phenomenon becomes less pronounced.

In addition to crosslink density, the T_50_ value is another characteristic parameter measured by TSSR (see Table 8). Specifically, it denotes the temperature at which stress decays to 50%. This parameter reflects the thermal stability of the network structure. Compared to the reference value, compounds containing devulcanized EPDM exhibit a reduced T_50_ value, coupled with a leftward shift of the relaxation peak, indicating diminished thermal stability of the crosslinked structure.

**Fig. 5:**
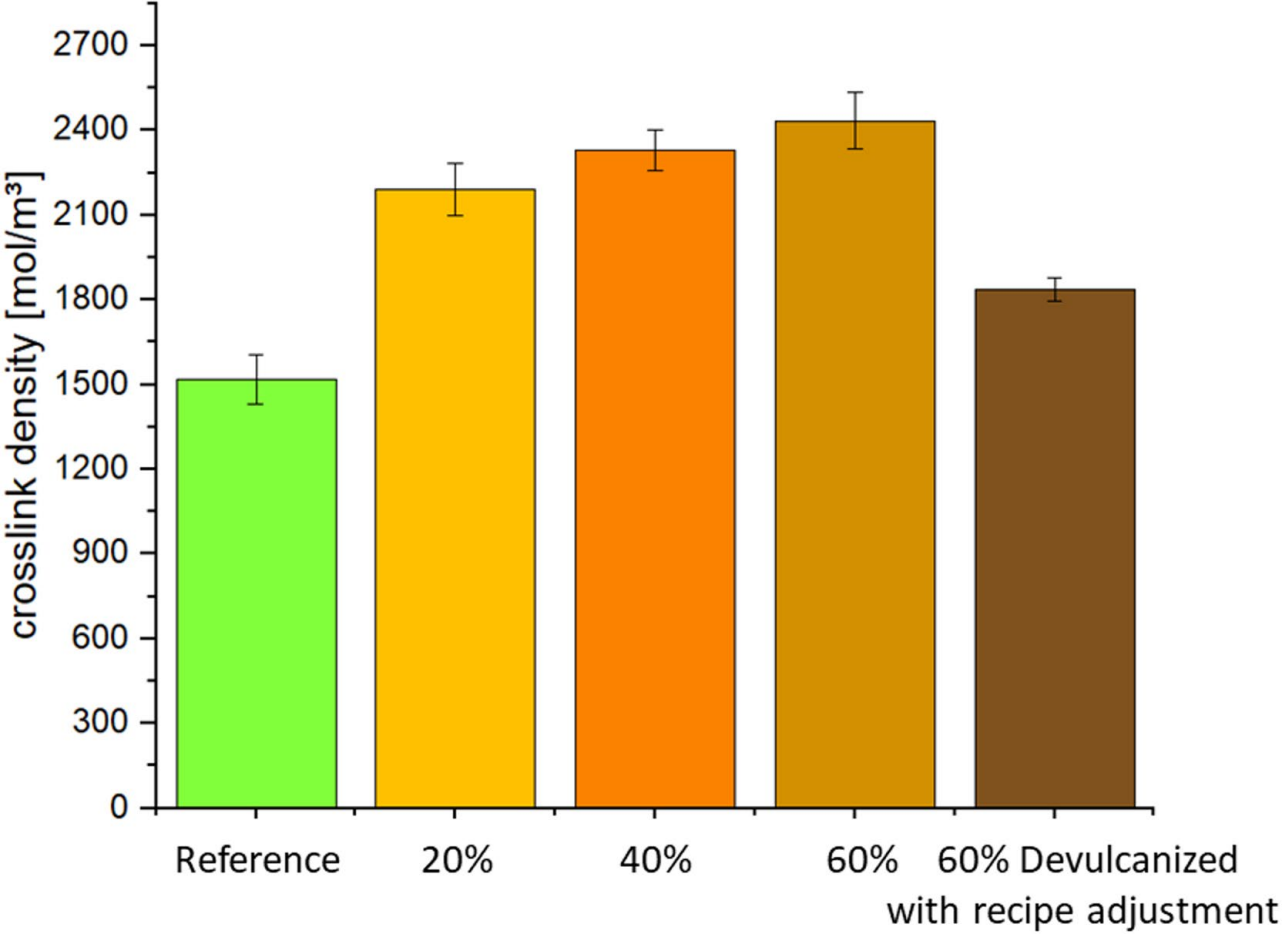
Determination of crosslink density via equilibrium swelling.

The equilibrium swelling method, based on the Flory-Rehner model^29^, determines crosslink density by measuring the volume change of a rubber sample immersed in solvent until it reaches a state of no further expansion. The expansion degree used to calculate crosslink density correlates with the polymer content of the sample—that is, after expansion and subsequent drying, the weight of the polymer network is determined by subtracting as much as possible the mass of fillers and other non-expanding components. Higher crosslink density results in lower swelling, as the mobility of the crosslinked network is more restricted. The results in Fig. 5 show that crosslink density increases with the content of devulcanized EPDM. However, the sample containing 60% devulcanized EPDM with recipe adjustments, exhibited lower crosslink density than other devulcanized EPDM-containing samples, yet it remained higher than the reference value. This indicates that polymer absorption of liquids also depends on the polarity of both the liquid and the polymer, as well as their mutual interactions. Polar polymers exhibit better resistance to nonpolar liquids but poorer resistance to polar liquids. Overall, the results demonstrate that the addition of devulcanized EPDM has a reinforcing effect, consistent with the TSSR findings.

Although most literature suggests that devulcanization reduces crosslink density, this paper presents differing results, which may be related to measurement methods. Furthermore, devulcanized rubber often contains residual free sulfur and partially broken sulfur crosslinks, components that retain reactivity. When devulcanized rubber is mixed with fresh compound and vulcanized, these residual sulfur compounds may trigger additional or uncontrolled crosslinking reactions. Consequently, increasing the proportion of devulcanized rubber often elevates overall crosslink density, as more active sites become available for forming new sulfur bridges during vulcanization^30^.

#### Mechanical properties

**Fig. 6:**
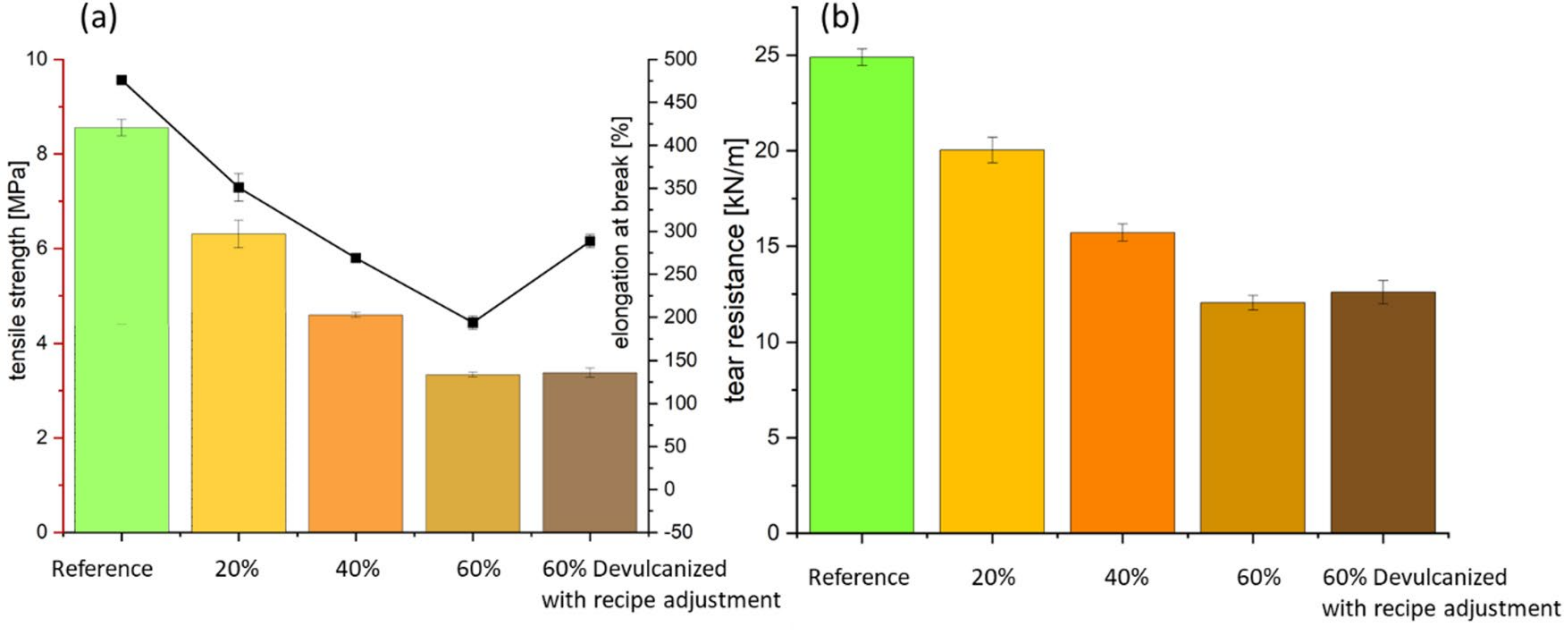
(a) tensile strength (solid bar), elongation at break (line graph); (b) tear resistance.

Figure 6 illustrates the effect of devulcanized EPDM content on the tensile properties and tear resistance of the compounds. As shown in (a), both tensile strength and elongation at break decrease with increasing devulcanized EPDM content, falling below the reference values. The compound containing 60% devulcanized EPDM with recipe adjustment exhibits higher elongation at break compared to others after formulation adjustment, yet it remains below the reference value. The tear strength results (b) follow the same trend as tensile strength.

Although crosslink density is crucial for good mechanical properties, excessive, irregular, or uneven crosslinking can adversely affect mechanical performance. This explains why the mechanical properties of samples containing devulcanized EPDM are inferior to the reference. For instance, crosslinks formed by residual sulfur may be unevenly distributed, creating regions of dense crosslinking adjacent to areas with sparse or damaged polymer chains. Under stress, this heterogeneity causes stress concentration, reducing tensile strength and elongation. Excessive crosslink density also diminishes chain mobility, making the material harder and more brittle. This brittleness diminishes impact resistance and elongation at break, increasing fracture likelihood under mechanical stress. The devulcanization process partially degrades the polymer backbone. Even with high crosslink density, shorter or damaged polymer chains contribute less to load-bearing capacity, thereby weakening the material’s overall strength.

**Fig. 7:**
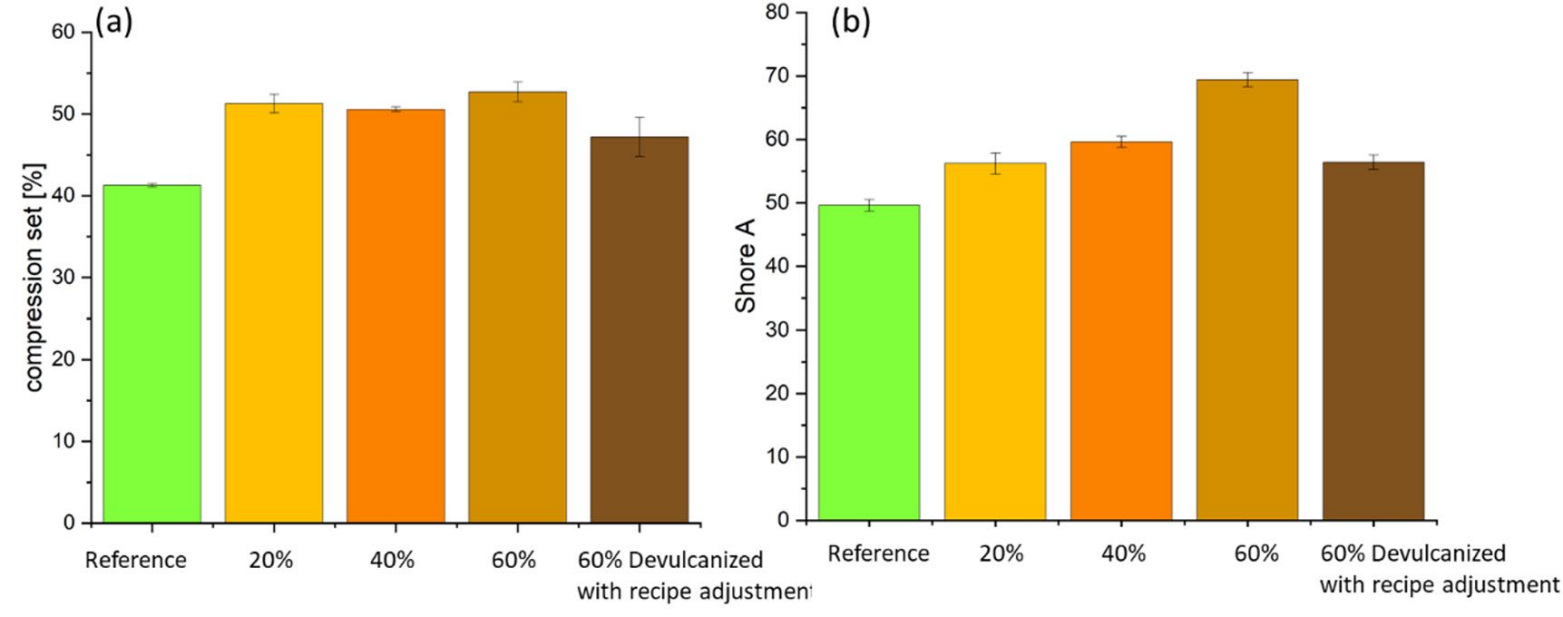
(**a**) Compression set 22 h at 100 °C; (**b**) Shore A hardness.

Compression set values measure the extent to which rubber components fail to recover to their original thickness after sustained compression at a specific temperature. As shown in Fig. 7(a), the addition of devulcanized EPDM increases compression set values, indicating poorer elasticity and deformation reversibility in the material. The decomposition and partial reorganization of the crosslinked network in devulcanized rubber leads to structural heterogeneity and imperfections in the polymer. As polymer chain mobility decreases or becomes increasingly restricted, the material loses some elastic recovery capability, reflected in the compression set. Samples containing 60% devulcanized EPDM exhibited the highest compression set values.

As shown in Fig. 7(b), the Shore A hardness increases with the content of devulcanized EPDM. However, the sample containing 60% devulcanized EPDM with recipe adjustments exhibited lower Shore A hardness, only slightly above the reference value. Residual crosslinking and re-crosslinking typically form a denser, harder network, thereby increasing material hardness, i.e., higher Shore A values. Recipe adjustment, however, exert a softening effect due to the compensation for carbon black.

#### Aging

**Fig. 8:**
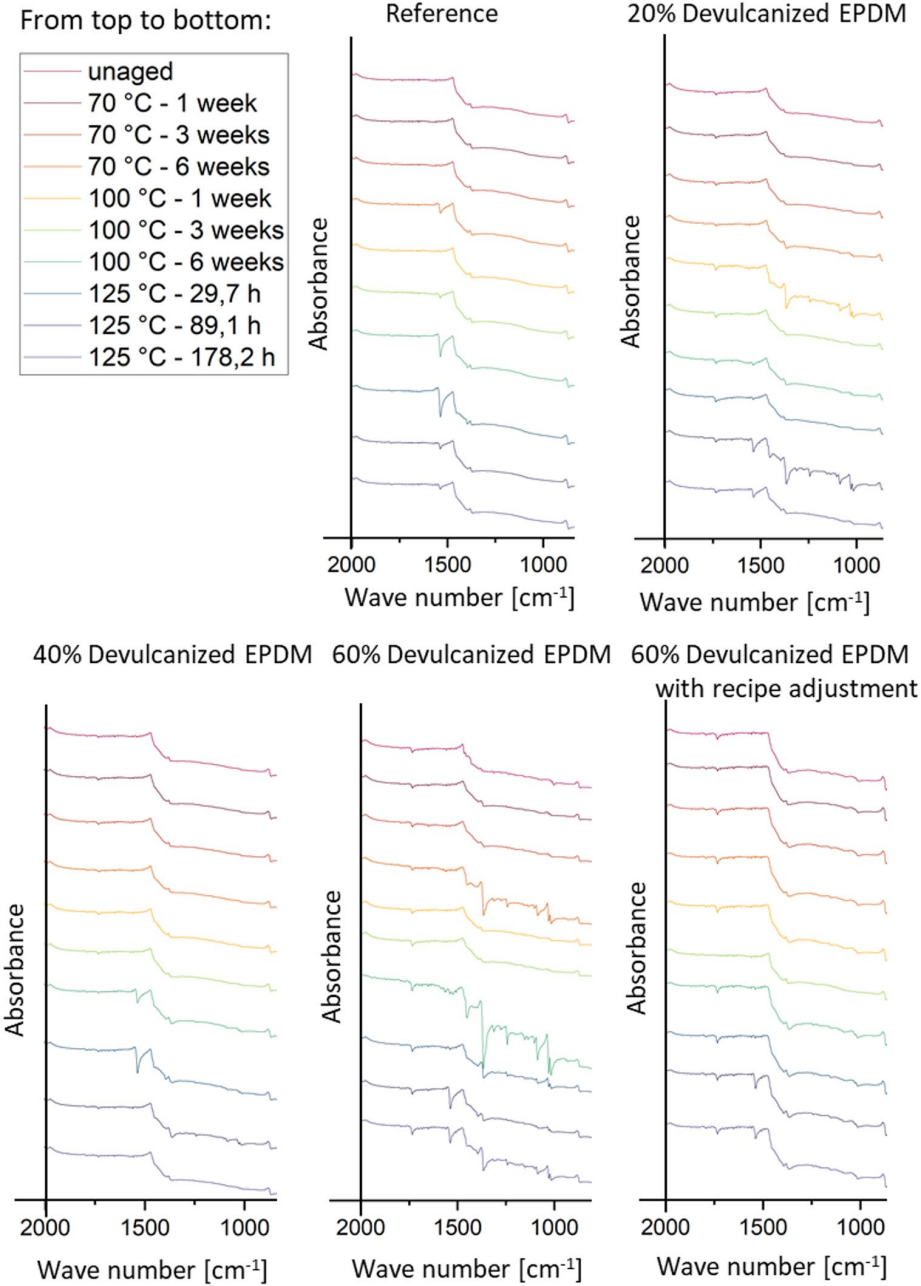
FTIR spectra of the reference, 20%, 40%, and 60% devulcanized EPDM (with recipe adjustment as well) compounds at different aging stages.

Figure 8 displays FTIR results for five samples at different aging intervals within the wave number range of 600 to 2000 cm⁻¹. When the content of devulcanized EPDM added was below 40%, the FTIR spectra showed no significant difference from the reference values. However, when the devulcanized EPDM content reached 60%, the spectrum at 6 weeks of aging (green) exhibited a marked difference. Increasing the content of devulcanized rubber typically leads to more pronounced aging effects due to the presence of residual sulfur, altered polymer chain stability, and changes in fillers. During aging, particularly oxidative aging, residual sulfur formed during devulcanization promote increased crosslinking or network rearrangement. Simultaneously, higher devulcanized rubber content introduces more vulnerable polymer chains, accelerating degradation processes such as chain scission and loss of elasticity. Nevertheless, recipe adjustment can substantially improve the aging behavior of samples, bringing them closer to the reference values.

**Fig. 9:**
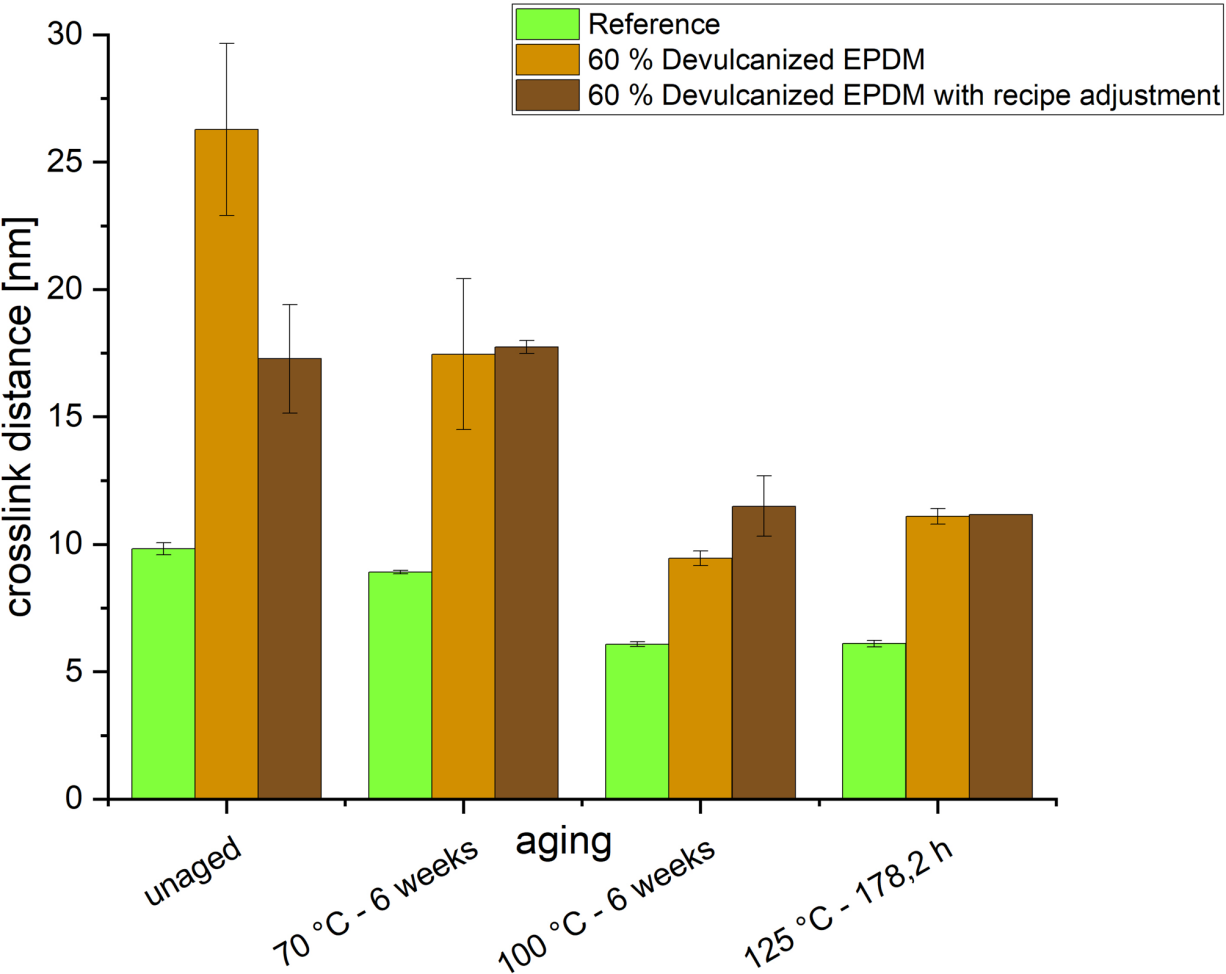
Change in crosslink density during aging of reference, 60% devulcanized EPDM without and with recipe adjustment compounds using freezing point depression method.

The crosslink distance describes the average spacing between two adjacent crosslinks in a polymer network and is inversely proportional to crosslink density. Figure 9 shows changes in crosslink distance measured using the freezing point depression method for unaged samples and at three temperatures during maximum aging duration including reference, 60% devulcanized EPDM rubber without recipe adjustment compounds, and with recipe adjustment compounds.

In the unaged specimens, the crosslink distance decreased in the following order: the compound containing 60% devulcanized EPDM, the compound containing 60% devulcanized EPDM with recipe adjustment, and the reference. This is because devulcanized rubber exhibits a larger initial average crosslink distance due to partial crosslink breakage during the devulcanization process. After aging, the crosslink distance decreased in all compounds, with a particularly significant reduction in the compound containing 60% devulcanized EPDM. After 6 weeks at 100 °C, the compound containing 60% devulcanized EPDM reached its minimum crosslink distance. After 178.2 h at 120 °C, the crosslink distance of the compound containing 60% devulcanized EPDM with a recipe adjustment reached its lowest value. The reference compound exhibited nearly identical crosslink distances under both conditions, indicating a complete alteration of the network structure. Recipe adjustment improved crosslink distance in the unaged state but proved largely ineffective after high-temperature aging. High-temperature aging causes polysulfide crosslinks to transform into disulfide and monosulfide crosslinks. Compared to polysulfide crosslinks, these exhibit lower mobility, leading to increased crosslink density. Since the devulcanized compound contains more polysulfide crosslinks relative to the reference as seen in the TSSR and compression set results, the difference in crosslink distance among the three samples after aging diminishes^31^.

**Fig. 10:**
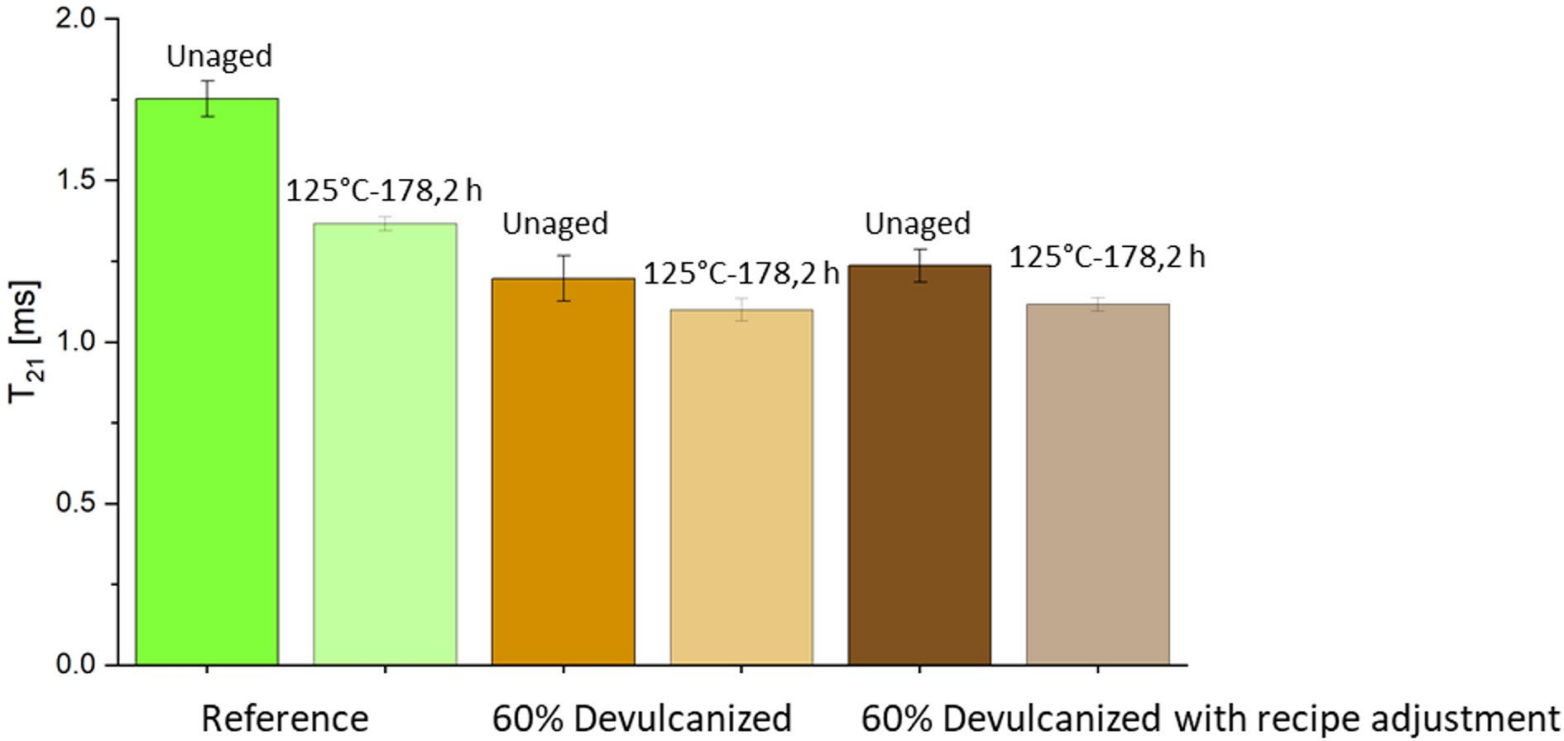
T_21_ relaxation time for the unaged and most aged samples of reference, 60% devulcanized EPDM without and with recipe adjustment compounds.

The T₂₁ relaxation time represents the spin-spin relaxation time obtained through low-field nuclear magnetic resonance (NMR) measurements, reflecting the migration capability of protons within the material. Specifically, the T₂₁ relaxation time corresponds to the timescale over which a nuclear spin loses coherence due to interactions with its local molecular environment. A shorter relaxation time indicates lower polymer mobility, thus it corresponds to higher crosslink density. In the unaged state, the compound containing 60% devulcanized EPDM exhibited the lowest migration rate, thus the highest crosslink density, followed by the recipe adjustment samples and the reference material, as shown in Fig. 10. This result aligns with equilibrium swelling (Fig. 5) but contradicts freezing point depression (Fig. 9). Aged samples exhibit significantly lower T_21_ values than unaged samples, with reduced variation between samples. This indicates aging increases crosslink density due to shifts from polysulfidic bonds to disulfidic and monosulfidic bonds, along with further crosslinking during aging.

The effects of aging on other mechanical properties are shown in Table 9. Overall, aging reduces the performance of all compounds, though the extent of reduction is less pronounced than for the reference material. Higher aging temperatures accelerate the rate and severity of mechanical property degradation. The Shore A hardness of different compounds exhibited varying trends at different temperatures prior to reaching one week of aging. The reference compound and the compound containing 20% devulcanized EPDM showed increased hardness at all temperatures. The compound containing 40% devulcanized EPDM exhibited increased hardness at 70 °C but decreased hardness at other temperatures. Compounds containing 60% devulcanized EPDM exhibited decreased hardness across all temperatures, with samples from the five modified formulations showing greater hardness reduction. Nevertheless, at one week of aging, the Shore A hardness of the samples still largely followed the unaged hardness sequence. The initial hardness reduction in EPDM compounds containing higher levels of desulfurized material may stem from chain defects and residual free sulfur in the high proportion of recycled material, accelerating chain breakage and network disruption. Modified formulations may mitigate this effect due to differences in filler dispersion or additive interactions. After one week of aging, hardness increased with rising temperature and extended aging duration. However, in the final stage, the differences between the five samples gradually diminished. The crosslinking and degradation reactions induced by aging tend toward equilibrium over extended periods, thereby essentially maintaining hardness ranking among the different compounds.

**Table 9:**
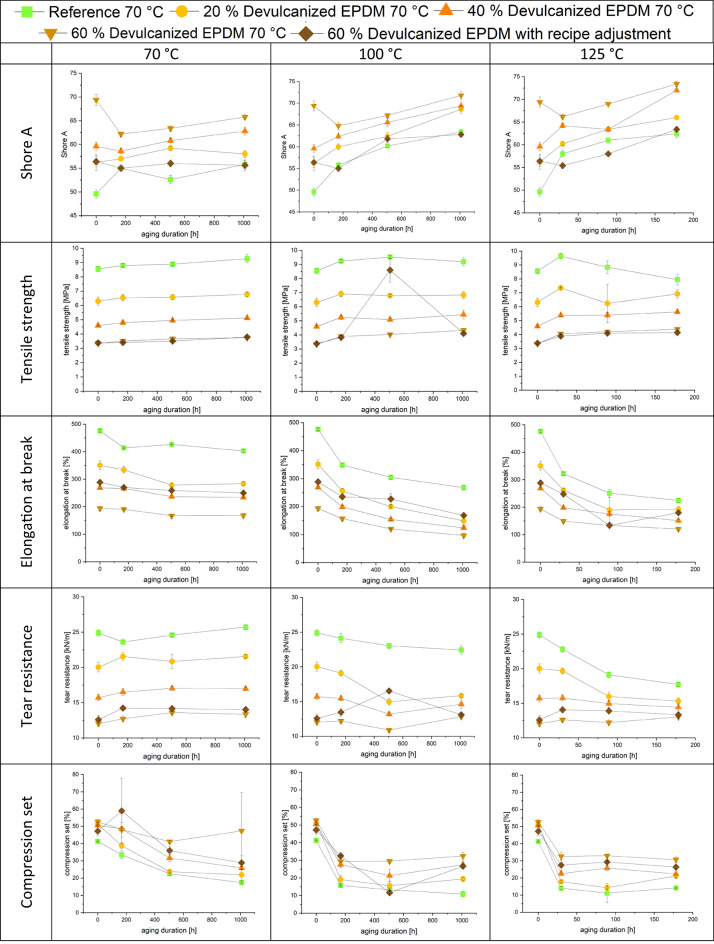
Mechanical properties after aging

Tensile properties indicate that the tensile strength of the reference compound increased throughout all durations at 70 °C, after 1 week and 3 weeks at 100 °C, and after 29.7 h at 125 °C, followed by a subsequent decline in performance. Compounds containing devulcanized EPDM exhibited similar trends, but their tensile properties began declining earlier than the reference after 1 week of aging at 100 °C, subsequently stabilizing. Compounds containing 60% devulcanized EPDM with recipe adjustment showed a peak value after 3 weeks of aging at 100 °C. The author suggests that the possible reasons may be that the additives introduced in the reformulated compound exhibit a certain degree of delayed degradation at 100 °C, or promote filler dispersion and matrix-filler interactions. These factors can inhibit premature segment breakage, allowing the post-curing effect to dominate during the mid-stage of aging. However, continued exposure to a thermo-oxidative environment will trigger segment breakage and oxidative degradation, ultimately leading to deterioration of the network structure and a progressive reduction in tensile strength as aging progresses. Elongation at break decreased with aging at all temperatures and time points.

The tear strength of the samples decreased during the aging process at 125 °C. However, starting from the first week at 70 °C and the third week at 100 °C, the tear strength largely recovered after the initial decline. The compound containing 60% devulcanized EPDM with recipe adjustment exhibited extremely high values again during the third week at 100 °C. Compression set decreased for nearly all compounds but stabilized after short-term aging at 100 and 125 °C due to changes in crosslinking during aging. Similarly, the compound containing recipe adjustment 60% devulcanized EPDM exhibited extremely low values again during the third week at 100 °C.

## Discussion

Five methods were employed in this study to measure the crosslink density of the compounds, or to indirectly compare variations in crosslink density, but the results were inconsistent. First, the maximum torque S’_max_ and torque increment ΔS’ measured using a rheometer in the curing performance (Table 7) showed values for compounds containing devulcanized EPDM that exceeded the reference values. This indicates enhanced overall network strength in the mixture, i.e., increased crosslink density. Secondly, the TSSR method can only measure the crosslink density of reference materials, limiting its application to compounds containing devulcanized EPDM. Due to residual free sulfur, polymer chain degradation, and changes in the crosslink structure, the thermal stability of devulcanized EPDM is typically lower than that of the original rubber. During TSSR testing, specimens are subjected to elevated temperatures and sustained strain to evaluate stress relaxation behavior. Under these conditions, devulcanized EPDM compounds may undergo further thermal degradation or physical aging during testing, leading to inconsistent stress relaxation responses. However, the significant increase in initial stress σ₀ relative to the reference value, as shown in Table 7, indirectly indicates enhanced crosslink density. Third, the equilibrium swelling method is a simple and widely applied technique for measuring crosslink density. Figure 5 also demonstrates that devulcanized EPDM leads to increased crosslink density. However, this method assumes a uniform network structure and ideal interactions between the elastomer and solvent. Swelling is also influenced by fillers, as fillers and crosslinking sites jointly determine the swellable volume within the solid matrix^32^. For devulcanized rubber, this may underestimate the polymer’s true swelling potential, thereby overestimating the crosslink density.

When studying aging behavior, changes in crosslink density measured via freezing point depression yield different results: 60% devulcanized EPDM exhibits increased crosslink spacing, i.e., reduced crosslink density (Fig. 9). This method is suitable for heterogeneous or complex systems but is sensitive to solvent-polymer interactions and impurities. Although the measured results correlate with changes in mechanical properties, they do not align with findings from other measurement techniques. It was suggested that the longer crosslink distance might result from a higher number of polysulfide bridges in devulcanized EPDM. However, further investigation is required. Compared to the expansion method, T_21_ relaxation time is less affected by filler content. Figure 10 shows consistent results with the previous three measurement methods, indicating that the addition of devulcanized EPDM leads to increased crosslink density.

In this study, an additional recipe adjustment was performed on the compound containing 60% devulcanized EPDM, specifically by reducing the proportions of carbon black and softener. Results indicate that this adjustment reduces Shore A hardness and increases the fracture enhancement rate, while other mechanical properties remain comparable to the unadjusted compound. Although different crosslinking density results were obtained using various measurement methods, this study considers the NMR relaxation time method more reliable. Therefore, the recipe adjustment can increase crosslinking density to some extent and improve structure. However, during aging behavior, several measurements for samples with the recipe adjustment showed outliers. Further replication is needed to verify whether this result is an anomaly or a genuine improvement in performance.

## Conclusion

This study comprehensively investigates the effects of incorporating varying amounts of devulcanized EPDM into an EPDM sealing compound. First, rheometer measurements of curing behavior indicate that devulcanized EPDM accelerates curing and increases crosslink density. Measurements based on TSSR, equilibrium swelling, and relaxation time also reveal the same trend. However, crosslink distance measured via freezing point depression indicates that increasing the proportion of devulcanized EPDM reduces crosslink density. This aligns better with the accompanying mechanical property changes but differs from the other four measurements. The authors speculate this may result from increased polysulfide bridges in devulcanized EPDM, though further research and validation are needed. These structural changes are critical as they directly impact the mechanical properties and durability of the resulting compounds.

Mechanical property evaluations reveal that higher devulcanized EPDM ratios correlate with increased shore A hardness, likely due to residual crosslinking and the inherently higher filler content in recycled rubber. Conversely, tensile strength and elongation at break decrease with increased recycled content. Thermo-oxidative aging assessments indicate that incorporating devulcanized EPDM does not accelerate degradation or cause premature embrittlement of the compound, performing comparably to virgin reference materials.

Furthermore, formulation optimization partially mitigated the negative effects of devulcanized rubber on crosslink density and flexibility, with minimal impact on other properties. These findings support the development of recycled rubber compounds with enhanced structural and functional characteristics, offering insights for improving the applicability of devulcanized recycled rubber across diverse applications.

## Data Availability

The datasets used and/or analysed during the current study available from the corresponding author on reasonable request.

## References

[1] Dorigato, A., Rigotti, D. & Fredi, G. Recent advances in the devulcanization technologies of industrially relevant sulfur-vulcanized elastomers. *Adv. Industrial Eng. Polym. Res.* **6**, 288–309 (2023).

[2] Warner, W. C. Methods of devulcanization. *Rubber Chem. Technol.* **67**(3), 559–566 (1994).

[3] Asaro, L. et al. Recycling of rubber wastes by devulcanization. *Resour. Conserv. Recycl.* **133**, 250–262 (2018).

[4] Joseph, A. et al. The current status of sulphur vulcanization and devulcanization chemistry: devulcanization. *Rubber Sci.* **29**, 62 (2016).

[5] Seghar, S. et al. Thermo-mechanical devulcanization and recycling of rubber industry waste. *Resour. Conserv. Recycl.* **144**, 180–186 (2019).

[6] Garcia, P. S., Sousa, F. D. B. & de Devulcanization of ground tire rubber: physical and chemical changes after different microwave exposure times. *Express Polym. Lett.* **9**, 1015–1026 (2015). Nr. 11LimaJ. A.de.

[7] Isayev, A. I. et al. Ultrasonic devulcanization of waste rubbers: experimentation and modeling. *Rheol. Acta*. **35**, 616–630 (1996).

[8] Tatangelo, V. et al. Biological devulcanization of ground natural rubber by Gordonia desulfuricans DSM 44462(T) strain. *Appl. Microbiol. Biotechnol.* **100**, 8931–8942 (2016). 27368738 10.1007/s00253-016-7691-5

[9] Tao, G. et al. The effect of devulcanization level on mechanical properties of reclaimed rubber by thermal-mechanical shearing devulcanization. *J. Appl. Polym. Sci.* **129** (Nr. 5), 2598–2605 (2013).

[10] Mnyango, J. I. et al. Effect of Tulbaghia violacea -Derived devulcanized rubber on the properties of natural rubber/Styrene‐Butadiene rubber blends. *Macromol. Mater. Eng.* **309**, 8 (2024).

[11] Mangili, I. et al. Characterization and supercritical CO2 devulcanization of cryo-ground tire rubber: influence of devulcanization process on reclaimed material. *Polym. Degrad. Stab.* **102**, 15–24 (2014).

[12] Tapale, M. & Isayev, A. I. Continuous ultrasonic devulcanization of unfilled NR vulcanizates. *J. Appl. Polym. Sci.* **70** (Nr. 10), 2007–2019 (1998).

[13] Kumnuantip, C. & Sombatsompop, N. Dynamic mechanical properties and swelling behaviour of NR/reclaimed rubber blends. *Mater. Lett.* **57**, 3167–3174 (2003).

[14] Maridass, B. & Gupta, B. R. Effect of extruder parameters on mechanical properties of revulcanized ground rubber tire powder. *Polimery* **52**, 456–460 (2007).

[15] Sabzekar, M. et al. Influence of process variables on chemical devulcanization of sulfur-cured natural rubber. *Polym. Degrad. Stab.* **118**, 88–95 (2015).

[16] Pirityi, D. Z., Bárány, T. & Pölöskei, K. Recycling of EPDM rubber via thermomechanical devulcanization: batch and continuous operations. *Polym. Degrad. Stab.* **230**, 111014 (2024).

[17] Heczko, J., Kottner, R. & Kossa, A. Rubber ageing at elevated temperature — Model calibration. *Eur. J. Mech. A. Solids*. **89**, 104320 (2021).

[18] Mostafa, A. et al. The influence of CB loading on thermal aging resistance of SBR and NBR rubber compounds under different aging temperature. *Mater. Design*. **30** (Nr. 3), 791–795 (2009).

[19] Rezig, N. et al. Thermo-oxidative ageing of a SBR rubber: effects on mechanical and chemical properties. *J. Polym. Res.* **27**(11), 339 (2020).

[20] Han, R. et al. Effects of crosslinking densities on mechanical properties of nitrile rubber composites in thermal oxidative aging environment. *J. Appl. Polym. Sci.* **137**, 36 (2020).

[21] Horikx, M. M. Chain scissions in a polymer network.. *J. Polymer Sci.* **19**(93), 445–454 (1956).

[22] Zhao, J. et al. Investigation of crosslinking in the thermooxidative aging of nitrile–butadiene rubber.. *J. Appl. Polymer Sci.*10.1002/app.41319 (2015).

[23] Jost, C. et al. Influence of thermo-oxidative aging on the structure and the water transport properties of sulfur-crosslinked EPDM. *Polym. Degrad. Stab.* **234**, 111210 (2025).

[24] Malissa, H. & Schmidts, W. Relative conductometric determination of carbon, hydrogen, oxygen, and sulfur. *Microchem. J.* **8** (Nr. 2), 180–193 (1964).

[25] Valentín, J. L. et al. Measurements of freezing‐point depression to evaluate rubber network structure. Crosslinking of natural rubber with Dicumyl peroxide. *J. Polym. Sci., Part B: Polym. Phys.* **45** (Nr. 5), 544–556 (2007).

[26] Basterra-Beroiz, B., Rommel, R., Kayser, F. & Into rubber network structure by a combination of experimental techniques. new insights. Rubber Chemistry and Technology 90 Nr. 2, S. 347–366. (2017).

[27] Bernal-Ortega, P. et al. Determination of the crosslink density of silica-filled styrene butadiene rubber compounds by different analytical methods. *Polym. Bull.* **81** (Nr. 1), 995–1018 (2024).

[28] Gschwind, L., Jordan, C. S. & Vennemann, N. Devulcanization of ethylene-propylene‐diene monomer rubber waste. Effect of diphenyl disulfide derivate as devulcanizing agent on vulcanization, and devulcanization process. *J. Appl. Polym. Sci.* **139**(20), 52141 (2022).

[29] Flory, P. J. & Rehner, J. Statistical mechanics of cross-linked polymer networks II swelling. *J. Chemical Physics* **11**(11), 521–526 (1943).

[30] Frosch, S. et al. Reduction in sulfur diffusion in recycled ground Rubber-Containing compounds to improve tensile strength. *Polymers* **17**, 21 (2025). 10.3390/polym17212942 PMC12608173 41228702

[31] Modrow, H. et al. Monitoring thermal oxidation of sulfur crosslinks in SBR-elastomers using sulfur K-edge XANES: a feasibility study. *Kautsch. Gummi Kunstst.* **63**, 328–337 (2000).

[32] Spanheimer, V. et al. Evaluation of the suitability of different methods for determination of the crosslink density in highly filled EPDM compounds. *J. Polym. Res.* **30** (Nr. 1), 1–10 (2023).

